# Length of stay in the emergency department and its associated input-, throughput-, and output factors at two hospitals in Sweden

**DOI:** 10.1186/s12873-025-01283-z

**Published:** 2025-07-15

**Authors:** Jonas Andersson, Lisa Kurland, Lena Nordgren, Annelie K. Gusdal, Ivy Cheng

**Affiliations:** 1 School of Medical Sciences, Örebro University, Campus USÖ, Örebro, 70182 Sweden; 2 School of Health, Care and Social Welfare, Mälardalen University, Box 325, Eskilstuna, 631 05 Sweden; 3 Department of Public Health and Caring Sciences, Centre for Clinical Research Sörmland, Uppsala University, Mälarsjukhuset, Eskilstuna, 631 88 Sweden; 4 Division of Emergency Medicine, University of Toronto, Sunnybrook Health Sciences Centre, 2075 Bayview Avenue, Toronto, ON M4N 3M5 Canada

**Keywords:** Emergency department, Length of stay, Explanatory factors, Patient flow

## Abstract

**Background:**

Prolonged emergency department length of stay (EDLOS) is a worldwide issue associated with increased mortality, decreased patient satisfaction and poor quality of care. The factors influencing EDLOS have not been comprehensively studied in the context of Swedish EDs. This study’s objective is to determine the input-, throughput- and output factors associated with EDLOS, at two urban EDs in Sweden.

**Methods:**

Data was collected from two hospitals. All patient visits during the two-year study period were included. Patients who left without being seen by a physician were excluded. The explanatory factors included patient characteristics, medical data, and hospital bed occupancy data. Multi-variable linear regression analysis was used to test the associations between the factors and EDLOS.

**Results:**

The top contributors to prolonged EDLOS were diagnostic imaging, which added between 64 and 149 min of EDLOS, diagnostic testing at central laboratory (53–99 min), followed by intra-ED zone transfer (46–94 min). Arriving during crowding or being admitted during high hospital bed occupancy had a significant but relatively small absolute effect on the outcome.

**Conclusions:**

Throughput factors had far greater impact on EDLOS than both input- and output factors. Adapting strategies to the structural and procedural characteristics of each setting may enhance the effectiveness of improvement efforts.

**Clinical trial number:**

Not applicable.

**Supplementary Information:**

The online version contains supplementary material available at 10.1186/s12873-025-01283-z.

## Background

Emergency Department (ED) Length of Stay (LOS) is the amount of time patients spend in the ED from arrival to discharge or admission [1]. Prolonged EDLOS is associated with increased mortality [2], decreased patient satisfaction [3] and poor quality of care [4]. This complex problem has been longstanding [5], worsening [6] and worldwide [7]. Different health jurisdictions have implemented national time-targets [8] and other clinical interventions [9], but improvements in wait times have been variable and unsustained [10].

The reason for prolonged EDLOS is complex and influenced by multiple factors [11], some of which are from processes in the ED, and some, such as boarding, are hospital and system issues [12]. A convenient way of organizing factors associated with EDLOS, is to apply Asplin’s input-throughput-output model [13]. This model was originally designed to identify components of the health care system that contribute to ED crowding, but the same components have been used to study patient flow and EDLOS [14].

Until 2017, Swedish EDs followed the international trend of increasing ED visits and longer LOS. However, since 2017, according to the Swedish Board of Health and Welfare [15], ED visits have decreased, but EDLOS has continued to increase [16]. A recent study based on data from Swedish ED visits in 2015–2019, suggests that patients admitted to in-hospital care may be more vulnerable to the effects of crowding. It also concluded that these effects are more prominent in counties with high levels of hospital bed occupancy [17]. Another Swedish study revealed that admitted patients spend more time in the ED, than patients discharged home, suggesting an output-related bottleneck to ED patient flow [18]. However, there are no Swedish studies investigating the impact of investigations, ex. laboratory testing and diagnostic imaging [19] or hospital occupancy [20]. A more comprehensive study of the factors associated with EDLOS is needed.

This study’s objective is to determine the input-, throughput- and output factors associated with EDLOS, at two urban EDs in Sweden.

## Methods

This study was designed as a cross-sectional observational study using data from electronic patient records. Since only routinely collected data was used, informed consent was waived. The study was approved by the Swedish Ethical Review Authority (DNR-2020-01614).

### Study setting

The study was conducted in two EDs, located in two separate administrative regions in central Sweden. Örebro University Hospital (OUH) has a catchment area of approximately 200,000 people and Mälarsjukhuset in Eskilstuna (MSE) has 120,000 people [21], but both EDs receive 50–65 000 visits annually [16]. Both hospitals were organized in a way that has been described in the literature as a “Hybrid model” [22], which refers to a dual authority, shared between the ED and other departments. Both OUH and MSE have 24-hour general surgery, anesthesiology, obstetrics, and pediatric coverage. OUH is a level 1 trauma center with an average of 402 beds during the study period, while MSE had 240 beds. Both EDs are organized with low proportions of physicians specialized in emergency medicine (EM), a newly established specialty in Sweden. A pre-triage nurse assesses patients and assigns them to the most appropriate medical specialty, based on the chief complaint. Within the ED patients are managed in separate zones, based on the specialty assignment made by the pre-triage nurse. Certain patient groups are redirected entirely from the ED and managed elsewhere. For example, psychiatric patients were not assessed in either of the studied EDs. For details on the case-mix and specific patient flow strategies for each hospital, see Appendix A. Both EDs used the Rapid Emergency Triage and Treatment System (RETTS) [23] as their main triage assessment tool.

### Study material

Two cohorts consisting of data extracted from consecutive electronic medical records of patients who visited the two EDs from January 1, 2018 to December 31, 2019 were included. The ED visit was defined as one patient’s continuous stay in the ED. The records of all ED patients, irrespective of age, were included. Observations of patients who left the ED without being seen (LWBS) were excluded.

### Data collection

The data sources included the ED patient registry, electronic health care records including laboratory and radiology records, and the Swedish population registry [24]. Demographics and ED process data, such as arrival time, were collected from the respective ED registries, but medical interventions, such as diagnostic imaging and laboratory tests, were retrieved separately from the respective hospital department data holdings and merged into the main dataset through time-stamps and personal ID numbers [24].

### Dependent variable

The primary dependent variable was EDLOS measured in minutes, defined as the time between patient arrival and patient departure, either by hospital admission or discharge home.

### Independent variables

**Fig. 1:**
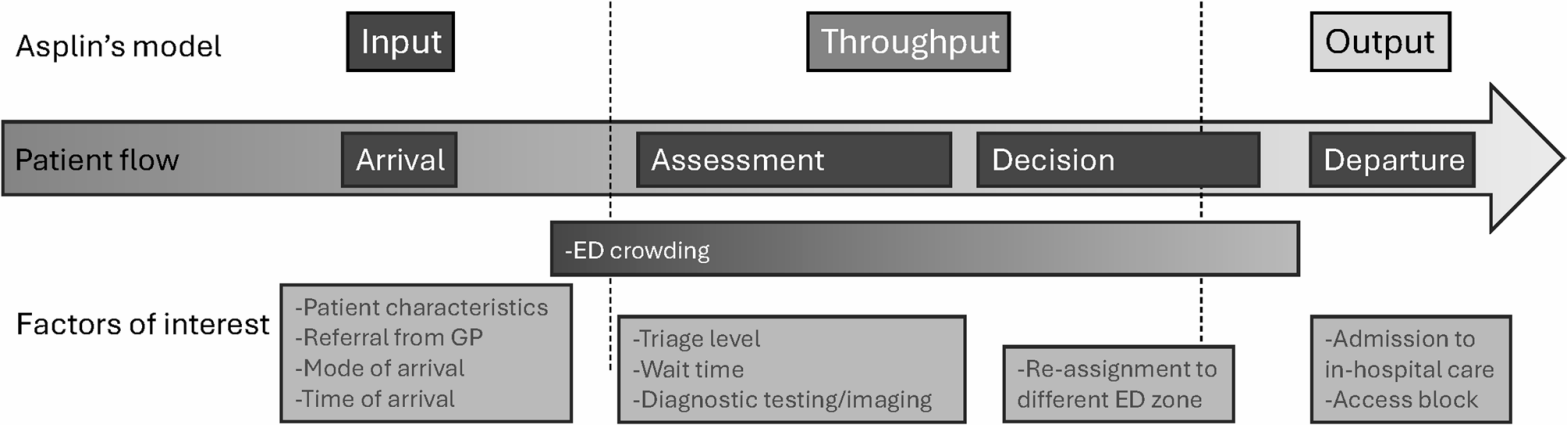
Patient flow in relation to Asplin’s model of emergency department crowding [13] and collected variables for the study

An outline of the independent variables collected and used in the regression analysis is provided below in the input-throughput-output model (Fig. 1). A detailed description of the independent variables is presented in Appendix B.

### Input factors

Patient demographics included sex (male/female) and age. Using the National Library of Medicine method of age grouping, age was categorized into children (0–17), adults (18–64), older adults (65–79) and elderly (80+) [25]. Other input factors collected were referral, mode of arrival and arrival time. *Referral* meant that upon arrival, the patient presented a written referral from a general practitioner or equivalent. *Mode of arrival* was either ambulance or walk-in. *Time of arrival* was categorized both by *day of week* and *hour of day*. After univariate regression analysis *day of week* was categorized into *Weekend* (Saturday and Sunday), *Monday* and *Other day*. Hour of day was categorized to correspond with personnel shift hours, which meant 8 AM − 4:59 PM, 5 PM − 9:59 PM and 10 PM − 7:59 AM.

### Throughput factors

*ED crowding* was measured using a model previously used in studies of ED crowding, conducted in settings similar to those used in the current study [17, 26]. The crowding exposure was defined as the mean ED census during the shift that the exposed patient arrived, divided by the expected census for the same shift. The expected census at each ED was estimated using a linear regression model with year, weekday/weekend, and arrival hour as predictors. The crowding exposure was categorized into three categories: low (0–75% of observations), moderate (76–95% of observations) and high (highest 5% of observations). *Triage level* was categorized into the five levels used by the RETTS acuity assessment [23] performed by the triage nurse. *Time-to-physician* was defined as time elapsed from arrival to first recorded contact with a physician.

Laboratory work was categorized to reflect different levels of analysis complexity. The specific analyses were chosen based on clinical experience as well as on the results of previous research [19]. The categories were: *no labs*; *labs excluding troponins and D-dimer*, meaning any sample of blood, spinal fluid or tissue collected and sent to the hospital’s central laboratory, but excluding observations where troponins or D-dimer was analyzed; *labs including troponins*,* not D-dimer*, were blood samples sent to central laboratory for troponin-I levels; *labs including D-dimer*,* not troponins*, were blood samples sent to central laboratory for D-dimer levels; *labs including D-dimer and troponins;* and *point-of-care testing only*, were samples of blood or urine that were analyzed only in the ED, without being sent to the central laboratory. Only recorded analyses performed during the ED stay were included.

Medical imaging was divided into: *X-ray* (plain imaging excluding interventional radiology, fluoroscopy, and angiography), *CT* (computed tomography) and *ultrasound* (sonographic non-interventional imaging, not bedside). *Other examinations* included magnetic resonance imaging, interventional radiology and other, to the ED context, rare types of examinations. Only examinations done within the ED stay were included.

*Intra-ED zone transfer* occurs when a patient is reassigned to a different specialty after being assessed by the index specialty assigned at pre-triage — e.g., when a patient presenting with abdominal pain is initially assigned to the surgical zone but transferred to the medical zone due to abnormal ECG findings. This transfer is not a physical transfer of the actual patient, but rather an administrative transfer and an indication that another medical specialty is medically responsible, while the patient remains in the ED.

### Output factors

Patient disposition was categorized into *discharged* or *admitted during low/moderate/high hospital bed occupancy.* Hospital bed occupancy was categorized as low (< 85%), moderate (85–95%) or high (> 95%). The Swedish National Board of Health and Welfare’s recommendation of 85% bed occupancy [27], and two other studies were used to determine the cut-off points. The two studies found that hospital bed occupancy above 95% was associated with increased rates of 30-day unplanned readmissions [28] and decreased rates of in-hospital admissions from the ED, suggesting that shortage of hospital beds could influence the ED physician’s decision to admit or discharge [29].

### Data cleaning and imputation

Data was assessed for quality, duplication and missing data. Duplicates and observations with poor data quality, such as negative duration for EDLOS or corrupted personal ID number, were removed. We considered EDLOS exceeding 48 h to be highly unlikely, thus indicative of data entry errors. Therefore, observations with EDLOS above 2880 min were considered outliers and removed from the data set. The cut-off was chosen based on clinical plausibility and supported by the empirical distribution of the data, where only a handful of observations exceeded this threshold. Because an intra-departmental transfer for the same patient visit generated a new encounter in the administrative database, these observations were merged into a single encounter. Multivariate imputation by chained equations was performed to estimate values for any variable with more than 5% missing values.

### Statistical analysis

Descriptive statistics were used for Table 1. A p-value less than 0.01 was considered to indicate statistical significance. For the primary outcome, a multivariable linear regression analysis was performed, using stepwise backward elimination technique to exclude nonsignificant covariates. The main analyses were performed for each hospital separately. An assessment of potential multi-collinearity was done using a Variance Inflation Factor (VIF) test (see Appendix C). Data was analyzed using Stata version 18 [30].

**Table 1:** Demographics: ED patient visits from 1 january, 2018 through 31 december, 2019, by study site

	Örebro university hospital (OUH)		Mälarsjukhuset Eskilstuna (MSE)	
Patient visits, n	123,730		98,317	
Age, mean (median)	41.9 (41.1)		45.5 (46.5)	
0–17, n (%)	32,588 (26.3)		20,201 (20.5)	
18–64, n (%)	56,337 (45.5)		47,966 (48.8)	
65–79, n (%)	21,550 (17.4)		18,607 (18.9)	
80+, n (%)	13,255 (10.7)		11,553 (11.7)	
Female sex, n (%)	60,874 (49.1)		49,857 (50.7)	
Arrival day, n (%)				
Monday	18,760 (15.2)		15,568 (15.8)	
Tuesday-Friday	69,694 (56.3)		56,000 (57.0)	
Weekend	35,276 (28.5)		26,759 (27.2)	
Arrival time of day, n (%)				
8:00 AM-4:59 PM	65,368 (52.8)		56,230 (57.1)	
5:00 PM-9:59 PM	34,332 (27.7)		24,937 (25.3)	
10:00 PM-7:59 AM	24,030 (19.4)		17,232 (17.5)	
Arrival by ambulance	24,792 (20.0)		19,203 (19.5)	
Referral ^a^, n (%)	9,991 (8.1)		1,982 (2.0)	
Top reasons for visit, n (%)	Abdominal pain 18,920 (16.3)		Abdominal pain 12,550 (14.6)	
	Dyspnea 8,575 (7.4)		Injury of wrist and hand 6,539 (7.6)	
	Chest pain 8,474 (7.3)		Dyspnea 6,503 (7.6)	
	Fever 7,118 (6.2)		Chest pain 5,669 (6.6)	
	Injury of head 5,703 (4.9)		Injury of head 4,115 (4.8)	
	Injury of wrist and hand 5,415 (4.7)		Injury of ankle and foot 3,608 (4.2)	
	Unspecified reason 4,314 (3.7)		Pain in limb 3,108 (3.6)	
	Infectious disease 3,042 (2.6)		Fever 3,004 (3.5)	
	Pain in limb 2,823 (2.4)		Dizziness and giddiness 2,331 (2.7)	
	Dizziness and giddiness 2,790 (2.4)		Injury of lower leg 2,134 (2.5)	
Crowding, observed/expected census ^b^	Observed mean (SD)	Expected mean (SD)	Observed mean (SD)	Expected mean (SD)
Low 0–75% ^c^	29.2 (11.4)	30.5 (10.0)	24.9 (9.9)	26.4 (8.6)
Moderate 76–94% ^c^	37.5 (14.9)	27.0 (10.8)	32.9 (12.9)	23.5 (9.2)
High 95–100% ^c^	29.3 (15.4)	16.0 (9.1)	26.1 (13.9)	14.2 (8.3)
Triage level, n (%)				
Red	5,202 (4.2)		2,418 (2.5)	
Orange	21,787 (17.6)		18,496 (18.8)	
Yellow	60,477 (48.9)		46,377 (47.1)	
Green	23,659 (19.1)		19,269 (19.6)	
Blue	12,603 (10.2)		11,767 (12.0)	
Median time-to-physician (IQR)	62 min (31–121)		59 min (26–119)	
Mean time-to-physician (SD)	89.2 min (82.3)		88.3 min (90.2)	
Laboratory work, n (%)				
No laboratory work	53,997 (43.6)		41,011 (41.7)	
Labs, excl. Troponins and D-dimer	41,346 (33.4)		41,190 (41.9)	
Labs, incl. Troponins not D-dimer	20,831 (16.8)		7,688 (7.8)	
Labs, incl. D-Dimer not Troponins	657 (0.5)		1,265 (1.3)	
Labs incl. Troponins & D-Dimer	1,176 (1.0)		1,128 (1.2)	
POC ^d^ analysis only	5,723 (4.6)		6,045 (6.2)	
Diagnostic imaging, n (%)				
No diagnostic imaging	92,192 (74.5)		60,328 (61.4)	
X-ray	11,413 (9.2)		20,766 (21.1)	
Ultrasound	2,223 (1.8)		2,691 (2.7)	
Computed tomography	17,654 (14.3)		14,542 (14.8)	
Other examination ^e^	248 (0.2)		0 (0)	
Intra-ED zone transfer, n (%)	4,574 (3.7)		3,508 (3.6)	
Patient disposition, n (%)				
Discharged home	90,479 (73.1)		73,384 (74.6)	
Admitted to ward during:				
Low bed occupancy (< 85%)	7,440 (6.0)		2,487 (2.8)	
Moderate bed occupancy (85–95%)	19,614 (15.9)		12,038 (12.2)	
High bed occupancy (> 95%)	6,197 (5.0)		10,490 (10.7)	
Hospital bed occupancy during study period, mean (SD)	89.4% (0.08)		93.3% (0.07)	
Patient outcomes				
Median EDLOS (IQR)	190 min (118–287)		203 min (125–309)	
Mean EDLOS (SD)	215.2 min (132.5)		231.5 min (147.5)	
EDLOS > 4 h, n (%)	43,886 (35.5)		39,084 (39.8)	
LWBS^f^, n (%)	1,593 (1.3)		3,168 (3.12)	
Deceased in ED, n (%)	118 (0.1)		92 (0.1)	
24-hour mortality, n (%)	348 (0.3)		293 (0.3)	

^a^ = Patient presented a referral from a general practitioner (or equivalent) upon arrival

^b^ = Observed mean census by shift– divided by expected mean census of the same shift, approximated by linear regression

^c^ = Crowding exposure categories based on the distribution of the quotient derived by the observed/expected census. Low = bottom 75% of observations, Moderate = 76–94% of observations, High = top 95% of observations

^d^ = Point-of-care

^e^ = Advanced diagnostic imaging excl. CT– e.g. Magnetic resonance imaging (MRI) or fluoroscopy

^f^ = Left the ED without being seen by a physician

## Results

**Fig. 2:**
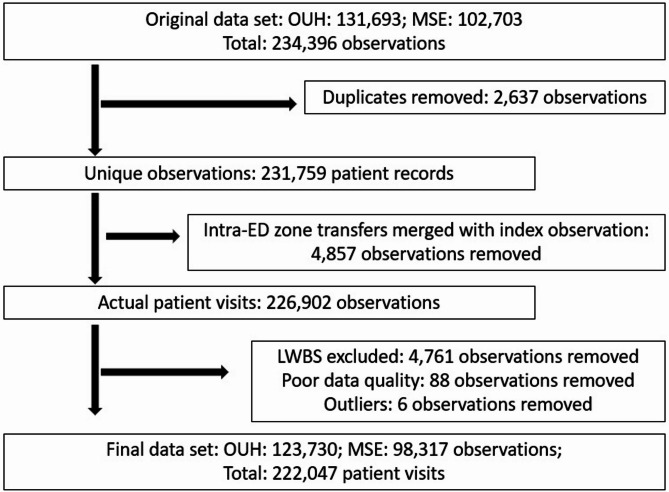
- Overview of data flow

After excluding duplicates and LWBS, 222,047 patient visits were included: 123,730 at OUH and 98,317 at MSE (Fig. 2). There were missing values for time-to-physician and triage level, 11.9% and 5.6% respectively. Missing values were estimated by imputation.

The median EDLOS for OUH and MSE were 190 and 203 min respectively. At OUH, the patients’ mean age was lower (41.1 years compared to 46.5 at MSE) and the proportion of pediatric patients (age < 18) was greater (26.3% compared to 20.5% at MSE). Abdominal pain was the most common reason for visit at both study sites. At MSE the mean hospital bed occupancy was higher (93.3% compared to 89.4%) and a higher proportion of patients were admitted during high bed occupancy (10.7 at MSE compared to 5.0% at OUH) (Table 1).

The linear regression analyses (Table 2) revealed that at OUH, all variables had a significant effect on the dependent variable EDLOS in minutes. At MSE, there were nonsignificant associations for arrival day of week and for being admitted during low hospital occupancy compared to being discharged home. The overall model fit was statistically significant (*P* < 0.01) with R^2^-estimates of 0.60 and 0.55 at OUH and MSE respectively.

**Table 2:** Linear regression analysis. Estimated coefficients and 95% confidence intervals (CIs) for associations with EDLOS

Independent variable	Coefficient (95% CI)	
	**Örebro University Hospital**	**Mälarsjukhuset Eskilstuna**
Age 0–17	11.8 (10.6–13.0) *	-8.3 (-10.1– -6.6) *
18–64 (reference)	0	0
65–79	7.6 (6.3–9.0) *	10.3 (8.6–12.1) *
80+	14.7 (13.1–16.4) *	11.3 (9.2–13.5) *
Sex, female	3.0 (2.1–3.9) *	2.7 (1.4–3.9) *
Arrival day		
Monday	2.0 (0.7–3.3) *	0.5 (-1.3–2.2)
Tuesday-Friday (ref.)	0	0
Weekend	-5.2 (-6.3– -4.2) *	0.8 (-0.6–2.3)
Time of day		
8:00 AM-4:59 PM (ref.)	0	0
5:00 PM-9:59 PM	-12.6 (-13.7– -11.5) *	-6.8 (-8.3– -5.3) *
10:00 PM-7:59 AM	-13.8 (-15.1– -12.6) *	-10.2 (-11.9– -8.5) *
Arrival by ambulance	19.3 (17.9–20.7) *	19.8 (18.0–21.6) *
Referral	2.9 (1.1–4.6) *	5.7 (1.3–10.1)
Crowding		
Low 0–75% (ref.)	0	0
Moderate 76–94%	5.4 (4.2–6.6) *	9.6 (8.0–11.1) *
High 95–100%	9.8 (7.6–11.9) *	11.5 (8.8–14.3) *
Triage level		
Red (ref.)	0	0
Orange	52.1 (49.6–54.7) *	64.2 (60.0–68.4) *
Yellow	38.6 (36.1–41.2) *	65.8 (61.5–70.0) *
Green	30.0 (27.2–32.7) *	57.7 (53.3–62.2) *
Blue	19.0 (16.0–22.1) *	52.6 (47.8–57.3) *
Time-to-Physician ^a^	0.9 (0.9, 0.9) *	0.9 (0.9, 0.9) *
Laboratory analysis		
No labs done (ref.)	0	0
Labs, excluding Troponins and D-dimer	61.1 (59.9–62.4) *	59.6 (58.0–61.3) *
Labs, incl. Troponins not D-dimer	52.8 (51.3–54.4) *	61.8 (59.2–64.5) *
Labs, incl. D-Dimer not Troponins	83.3 (76.9–89.7) *	68.3 (62.7–74.0) *
Labs incl. Troponins & D-Dimer	98.3 (93.5–103.1) *	98.7 (92.8–104.6) *
POC ^b^ analysis only	38.8 (36.5–41.1) *	26.0 (23.3–28.8) *
Diagnostic imaging		
No radiology (ref.)	0	0
X-ray	67.7 (66.0–69.4) *	63.8 (62.1–65.5) *
Ultrasound	148.5 (145.0–152.0) *	121.2 (117.4–125.1) *
Computed tomography	131.7 (130.3–133.1) *	125.3 (123.5–127.1) *
Other examination^c^	143.1 (132.8–153.4) *	-^c^
Intra-ED zone transfer	93.5 (90.9–96.1) *	46.4 (43.0–49.8) *
Disposition		
Discharged (ref.)	0	0
Admitted, < 85% hospital bed occupancy	3.5 (1.5–5.6) *	0.7 (-3.4–4.7)
Admitted, 85–95% bed occupancy	8.0 (6.5–9.4) *	4.5 (2.4–6.7) *
Admitted, > 95% bed occupancy	7.4 (5.2–9.7) *	16.5 (14.3–18.8) *
Intercept	28.2 (25.3–31.0) *	15.4 (10.9–20.0) *

* = Statistically significant association with the dependent variable, p-value < 0.01

^a^ = Continuous variable, unit = 1 min increments

^b^= Point-of-care

^c^= No “Other examinations” (i.e. MRI, fluoroscopy angiography etc.) were carried out during ED stay at MSE, the variable was omitted from the model

Arrival by ambulance was the largest contributor to increased EDLOS among the input factors at both study sites, and was associated with an increase of 19 min. Patients aged 65 and above were more likely to have an increased EDLOS at both study sites, compared to patients aged 18 to 64. Patient age < 18 was associated with an increased EDLOS (12 min) at OUH, but a decreased EDLOS (-8 min) at MSE. Arrival time during the evening- or night shift decreased the estimated EDLOS at both study sites.

For the throughput factors, diagnostic imaging was the greatest contributor to the estimated EDLOS at both study sites with non-bedside ultrasound adding 148 min at OUH and CT adding 125 min at MSE. Laboratory analysis including troponins and D-dimer increased EDLOS with 98 and 99 min at OUH and MSE respectively. Patients whose laboratory work included only POC-analysis had an estimated increase in EDLOS of 39 and 26 min at OUH and MSE respectively, compared to patients who had no laboratory work performed. *Intra-ED zone transfer* added 94 min to the estimated EDLOS at OUH and 46 min at MSE. *Moderate* (76–95% of all observations) *ED crowding* increased the estimated EDLOS with 5 and 10 min at OUH and MSE respectively.

For the output factors, being admitted to in-hospital care increased the estimated EDLOS at OUH by 4, 8 and 7 min, during low (< 85%), moderate (85–95%) and high (> 95%) hospital bed occupancy respectively. At MSE there was no significant effect of being admitted during low hospital bed occupancy compared to being discharged from the ED. At moderate and high bed occupancies, the estimated increases in EDLOS were 5 and 17 min respectively.

## Discussion

In this study of factors associated with EDLOS at two hospitals in Sweden, we found that the factors with the greatest impact were throughput related. Diagnostic imaging and laboratory testing surpassed the impact of input and output factors by a large margin. Intra-ED zone transfer was associated with significant and substantial increases in EDLOS estimated at 94 and 46 min at OUH and MSE respectively. This effect is, to our knowledge, the first recorded estimation of the impact of a Hybrid model of organizing emergency departments [22] and both the magnitude of the effect, as well as the difference between study sites, highlights the importance of ED organization regarding authority and patient responsibility. The effect of hospital bed occupancy on EDLOS was more prominent in the hospital with fewer hospital beds and higher mean bed occupancy. While there were similarities between the study sites, our study also revealed important differences in the effects of several factors, which highlights the uniqueness of each ED.

At both study sites, older age was associated with an increased EDLOS, compared to patients aged 18 to 64. Similar results have been seen in previous studies and can most likely be attributed to higher patient complexity [18, 31]. Female sex was also associated with a small but significant increase in EDLOS, which is not as easily explained by complexity and not as frequently reported. This association has, however, been described previously [18], but the underlying cause remains to be investigated. Young age (< 18 years) was associated with increased EDLOS at OUH, but with decreased EDLOS at MSE. These contrasting findings may reflect the organizational differences between the study sites. Specifically, MSE lacked a designated area tailored for children, which may have contributed to a tendency to expedite the disposition of pediatric patients [32]. Alternatively, the finding is reflective of a cultural difference of the study sites. OUH, seeing more pediatric patients, might be more prone to time-consuming practices of alleviating pain and distress associated with common procedures [33].

Laboratory analysis and diagnostic imaging were associated with significant increases in EDLOS, ranging between one and two hours of added time across both study sites. These results are in line with previous research and therefore not surprising [20]. Laboratory testing, depending on complexity, was associated with additional EDLOS between 55 and 93 min, which can be compared to an overall effect of 72 (95% CI: 66–78) minutes for blood testing, estimated in a large nationwide study in United States [34]. Advanced imaging (ADI), including CT and MRI, was estimated to increase EDLOS by 121–143 min, which can be compared to the results of another study using the United States National Hospital Ambulatory Medical Care Survey, who found ADI to be associated with an increased EDLOS ranging between 47 and 123 min, depending on patient symptoms. For abdominal pain, the most common complaint in our study, the 95% CI was 97–141 min [35]. The amount of additional time associated with diagnostic testing and imaging, suggests that alternative methods, such as point-of-care (POC) ultrasound [36] and POC-testing [37] should be given careful consideration in clinical practice. Our results should also be interpreted in the light of several studies claiming that a substantial amount of diagnostic imaging is deemed inappropriate [38–40].

There was a moderate overall effect of ED crowding. A high level of crowding was associated with an increased EDLOS of 10 and 11 min, at OUH and MSE respectively. However, this effect was observed only in the top 5th percentile of observations. For the majority of the ED population at OUH, crowding was associated with 5 min of additional EDLOS or less. These results contrast with previous research [18, 41], where crowding has been identified as a main contributor to prolonged EDLOS globally. In another study conducted in Sweden, crowding was associated with increases in EDLOS ranging between 45 and 117 min, depending on chief complaint [42]. One possible explanation for this difference, is that none of the abovementioned studies has taken the possible effect of diagnostic testing and imaging into account. It is also plausible to assume that ED crowding has greater effect on staff workload [43] and patient safety [44], than it has on the length of stay for individual patients.

At both of the study sites, the output factors were substantially smaller than most throughput factors, which contradicts the results from a large overview in North America [45] as well as the conclusions of an international systematic review [7]. This can partly be explained by the relatively moderate rates of bed occupancy during the study period. It is, however, important to note that the data for this study were collected before the COVID-19 pandemic. Since then, the total number of hospital beds in Sweden has decreased [46], and the number of patients admitted to wards already at maximum capacity has increased [47]. It is also noteworthy that the median EDLOS for both EDs in this study was approximately three hours. This was somewhat below the national average for Swedish EDs and significantly below the median EDLOS in more densely populated regions such as Stockholm and Gothenburg [15].

### Strengths and limitations

A strength of this study was the use of robust data from multiple sources and a large sample size. There were missing data that required imputation; however, the absence of significant changes following imputation implied that the data were robust. Although this study benefits from robust data and a large sample size, it has several limitations. The generalizability of our findings may be limited by the specific settings in which the study was conducted. Also, the use of EDLOS in its continuous form as the dependent variable challenges the assumption of normality for linear regression with ordinary least squares (OLS). However, the standardized residuals of the predicted values were found to be normally distributed (see Appendix C) and even though a generalized linear model (GLM) might have strengthened the statistical integrity, a previous study using similar data has shown very similar results when comparing OLS and GLM [35]. The intuitive interpretation of estimated coefficients offered by OLS linear regression was ultimately decisive for the choice of statistical analysis.

Additionally, our study used the bed occupancy rate as a proxy for access block. Ideally, a direct measurement of the time patients spent waiting for a hospital bed would have been preferable. Unfortunately, the absence of data on bed-request time constrains our ability to provide more precise measurements in this regard. Another limitation is that a lot of time has passed between data collection and publication, which means that some of the explanatory factors may not still be applicable. One such factor is the hospital bed occupancy rate, which is likely to have increased substantially since the time of the data collection [46]. In addition, the choice of proxy measure for crowding can be seen as a limitation. Using a simpler and widely acknowledged measure like the *ED bed occupancy rate* [48], might have increased the interpretability of the results. However, it was decided, based on our own knowledge of the study sites and previous research at similar settings, to use a measure with better potential for capturing the effect of unexpected surges in patient flow [17, 26].

### Clinical implications

The study results can be used by ED management and health care policy makers, aiding in the prioritization of interventions aimed at reducing EDLOS. At OUH, the difference between being admitted during low and high hospital occupancy was less than 4 min, meaning that the effect of access block on EDLOS was very weak. Therefore, an attempt to increase the number of hospital beds might prove to be an expensive and inefficient intervention, if reduced EDLOS is the aim. Additionally, the large effect of complex laboratory testing and advanced diagnostic imaging supports the development of clinical pathways. Either with the aim to delay or refrain from a test– or to increase the use of alternative diagnostic methods, such as POC-sonography and -testing.

The large difference between the effects of intra-ED zone transfer observed at the two study sites suggests that the procedure of reassigning patients to other specialties has potential to be made more time efficient. Moreover, we suggest that each ED use its operational data to identify its unique bottlenecks to ED flow.

## Conclusions

In conclusion, this study highlights the substantial impact of throughput factors - particularly diagnostic imaging and intra-ED zone transfers - on emergency department length of stay (EDLOS). The findings underscore the importance of context-sensitive interventions that address operational inefficiencies contributing to prolonged EDLOS. Adapting strategies to the structural and procedural characteristics of each setting may enhance the effectiveness of improvement efforts.

## Electronic supplementary material

Below is the link to the electronic supplementary material.

Supplementary Material 1

## Data Availability

Unfortunately, the data set is not permitted to be available for open access. Data used for this study is sensitive patient data from Örebro University Hospital and Mälarsjukhuset Eskilstuna. These data are not publicly available due to restrictions governed by the Swedish Ethical Review Authority, and Swedish laws in relation to research in, and distribution of health care data. However, Örebro University researchers are mandated to archive research data for 10 years if an audit is required. To gain access to the data, please contact the Swedish Ethical Review Authority and cite registration number: 2020-01614.

## Abbreviations

ADI: Advanced diagnostic imaging
CI: Confidence interval
ECG: Electrocardiogram
ED: Emergency department
EM: Emergency medicine
EDLOS: Emergency department length of stay
GLM: Generalized linear model
IQR: Interquartile range
LOS: Length of stay
LWBS: Left without being seen (by a physician)
MSE: Mälarsjukhuset Eskilstuna (Hospital)
OLS: Ordinary least squares
OUH: Örebro university hospital
POC: Point-of-care
RETTS: Rapid Emergency Triage and Treatment System
SD: Standard deviation
VIF: Variance inflation factor
