# Supplementary material for: Length of stay in the emergency department and its associated input-, throughput-, and output factors at two hospitals in Sweden
Source: BMC Emerg Med. 2025 Jul 15;25:120. doi: 10.1186/s12873-025-01283-z (PMC12261713; doi:10.1186/s12873-025-01283-z)
Supplement: Supplementary file 1 — Supplementary Material 1 [file 12873_2025_1283_MOESM1_ESM.docx]

## Appendix A, Specification of patient in-flow at the study sites

Both EDs at the study sites had pre-triage nurses assigning incoming patients to the appropriate medical specialty, based on chief complaint (or referral from a general practitioner). The different patient categories were managed differently at the different hospitals. Some specialties had permanent on-call physicians at the ED, while others saw their patients at separate facilities. For some specialties the separate facilities only saw patients during office hours, while others saw patients at all times and days. This results in the redirection of patients at the door of the ED. The different medical specialties and the respective patient flow management for the different hospitals are listed below.

| **Subspecialty assignment** | **Örebro University Hospital** | **Mälarsjukhuset Eskilstuna** |
| --- | --- | --- |
| Internal medicine | ED | ED |
| General surgery | ED | ED |
| Orthopedics | Redirect minor injuries during office hours | ED |
| Pediatric internal medicine | ED (separate zone) | Redirect during office hours |
| Cardiology | ED | ED (managed by Internal medicine) |
| Infectious diseases | ED | ED (managed by Internal medicine) |
| Neurology | ED | ED (managed by Internal medicine) |
| Urology | ED | ED (managed by General surgery) |
| Ear-nose-throat | Redirect during office hours | Redirect during office hours |
| Gynecology | Redirect to external facility | Redirect during office hours |
| Ophthalmology | Redirect to external facility | Redirect to external facility |
| Psychiatry | Redirect to external facility | Redirect to external facility |

## Appendix B, Data merging, variable calculation and categorization

EDLOS was calculated by subtracting arrival time from departure time, in accordance with the definition supported by the International Federation for Emergency Medicine (1). Arrival time was first recorded contact at ED. On both study sites, this is done in close proximity to physical entry (door time), but some delay between actual “door time” and registration will inevitably be hidden outside the data. Departure time is also collected using administrative records, as the time stamp is generated by the electronic health record system, upon discharge or transfer to in-hospital ward.

Patient age was calculated using a function in the statistical software (30) returning age based on birth date and arrival date. Birth date was retrieved using the Personal identification number (2).

ED zone transfer: The internal transfers between different zones in the ED, all generated a new record in the ED patient registry. Thus, each index record was identified, and the subsequent record was merged with the index record. The departure time was adjusted to represent the actual patient visit. The procedure of merging observations was done using timestamps and personal identity numbers.

Referral from GP: This is a proxy measurement. The actual written referrals were not collected for this study, which means that we cannot be certain that the referral is actually written by a general practitioner. The variable is collected from the ED patient registry and indicates that someone (usually the pre-triage nurse) has marked the box “Kommer på remiss” (roughly translates to “Arrives on referral”) upon patient arrival. On rare occasions, the referral can have been written by a physician outside of the Swedish Primary health care system.

Triage level: RETTS is a 5-level scale using color coding to prioritize patients; red=immediate life threat, orange=potential life threat, yellow=urgent, green=not urgent and blue=limited need of emergency care. The assessment included the measurement of vital signs and an evaluation of the patient’s chief complaint and other related signs and symptoms (3). The chief complaint recorded at triage was collected to the main data set and used in this study for descriptive statistics and imputation of missing triage level values.

The hospital bed occupancy rate: This measurement was available for certain time-stamps each day; at 6 AM, 12 AM, 6 PM and 9 PM at OUH and at 6 AM, 6 PM and 9 PM at MSE. For each patient observation in the dataset, the hospital occupancy rate prior to the patient’s departure time was registered.

1. Hruska K, Castren M, Banerjee J, Behringer W, Bjornsen LP, Cameron P, et al. Template for uniform reporting of emergency department measures, consensus according to the Utstein method. Eur J Emerg Med. 2018.

2. Agency ST. Population registration in Sweden. 2007. Report No.: SKV 717B.

3. Widgren B. Rätt med RETTS beslutstöd - Utmaningar och möjligheter inom akutsjukvård. 2:nd ed. Göteborg: B4PRESS förlag; 2019.

## Appendix C – Missing data analysis, post-estimation and sensitivity analysis

There were missing data in the raw data set. For most variables, the number of missing data points were few enough, that the impact of those observations was considered negligible. However, for the *Triage level* and *Time-to-physician* variables, there were missing values in 5.6 % and 11.9 % of observations respectively. The observations with missing values in either of these variables had significantly shorter EDLOS and were to a greater degree admitted to in-hospital treatment, compared to the observations without missing values. We therefore concluded that the missing data could not be considered missing completely at random (MCAR). Missingness was, however, related to observed variables, such as EDLOS and disposition, and distributed randomly within groups defined by observed data. Therefore, missing data was assumed to be missing at random (MAR) – an assumption that was strengthened by the consequent sensitivity analysis (1). Since the variables with missing data could be related to one another, a Multiple Imputation with Chained Equations (MICE) approach was considered most appropriate. Triage level was estimated using Chief complaint, Time-to-physician, laboratory work, disposition and EDLOS. Time-to-physician was estimated using Triage level, ED occupancy rate and EDLOS. The chained equations were iterated 20 times.

Since R^2^ values differ across imputed datasets, we used a pooling method to obtain a single summary estimate. Each R^2^ value was first transformed using Fisher’s Z-transformation to stabilize variance. The transformed values were then averaged across all imputed datasets. Finally, the mean Z value was back-transformed to yield a pooled R^2^ estimate. This approach provides a statistically appropriate summary of model fit across multiple imputations (2). The models demonstrated good explanatory power, with adjusted R² values of 0.60 for OUH and 0.55 for MSE.

The dependent variable was not normally distributed. However, when analyzing the residuals predicted by the linear model, the deviations from normality and homoscedasticity were acceptable. See density plot and “Residuals vs fitted values”-plot below:


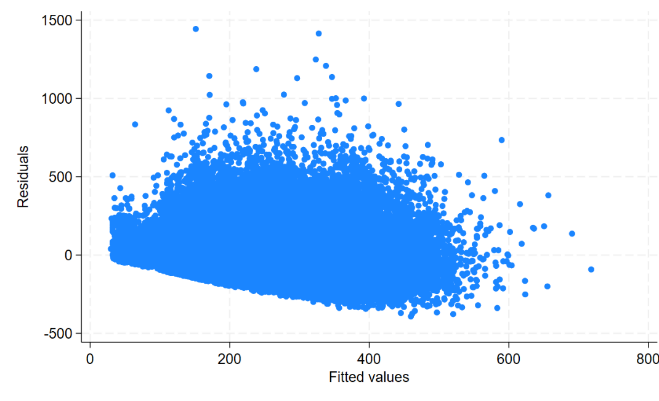


To assess potential multicollinearity, a variance inflation factor (VIF) test was carried out for the models at both study sites. A VIF of 5 or above for individual variables, or a mean VIF substantially greater than 1, was considered indicative of moderately strong multicollinearity (3). An overview of the variance inflation factors is provided below:

|  | **MSE** | | **OUH** | |
| --- | --- | --- | --- | --- |
| **Model** | Full model | No Triage | Full model | No Triage |
| Age0_17 | 1.3 | 1.3 | 1.35 | 1.31 |
| Age65_79 | 1.21 | 1.21 | 1.21 | 1.21 |
| Age80_plus | 1.26 | 1.26 | 1.24 | 1.24 |
| Gender | 1.01 | 1.01 | 1.01 | 1.01 |
| ArrDay |  |  |  |  |
| Monday | 1.11 | 1.11 | 1.09 | 1.09 |
| Weekend | 1.1 | 1.1 | 1.13 | 1.12 |
| ArrHour17_21 | 1.16 | 1.15 | 1.18 | 1.17 |
| ArrHour22_07 | 1.15 | 1.14 | 1.17 | 1.17 |
| ArriveAmb | 1.37 | 1.32 | 1.41 | 1.32 |
| Referral | 1.03 | 1.02 | 1.06 | 1.05 |
| Crowding |  |  |  |  |
| Medium | 1.05 | 1.05 | 1.05 | 1.05 |
| High | 1.05 | 1.05 | 1.04 | 1.04 |
| TriageLevel |  |  |  |  |
| 2 | 7.21 | - | 4.43 | - |
| 3 | 11.66 | - | 7.57 | - |
| 4 | 8.19 | - | 5.53 | - |
| 5 | 6.29 | - | 4 | - |
| TTD | 1.14 | 1.1 | 1.17 | 1.09 |
| Labs |  |  |  |  |
| 1 | 1.73 | 1.54 | 1.64 | 1.47 |
| 2 | 1.33 | 1.24 | 1.64 | 1.49 |
| 3 | 1.06 | 1.05 | 1.03 | 1.02 |
| 4 | 1.05 | 1.03 | 1.04 | 1.03 |
| 5 | 1.14 | 1.11 | 1.11 | 1.09 |
| XrayDone | 1.26 | 1.12 | 1.13 | 1.05 |
| USDone | 1.04 | 1.04 | 1.03 | 1.03 |
| CTDone | 1.11 | 1.11 | 1.12 | 1.11 |
| OtherExam | - | - | 1 | 1 |
| b_ChangeDept | 1.03 | 1.03 | 1.14 | 1.13 |
| Admitted |  |  |  |  |
| 1 | 1.07 | 1.06 | 1.16 | 1.14 |
| 2 | 1.27 | 1.22 | 1.36 | 1.31 |
| 3 | 1.25 | 1.21 | 1.14 | 1.12 |
|  |  |  |  |  |
| Mean VIF | 2.16 | 1.14 | 1.74 | 1.15 |

To test the robustness of the results - and to assess the potential impact of multi-collinearity in the models - different modifications to the model were made and the resulting estimations were compared. The potential multi-collinearity is likely due to correlation between *Time-to-physician* and *Triage level*. The sensitivity analysis has shown that *Triage level* could potentially be removed from the model, without sacrificing much explanatory power. However, keeping Triage level in the model did not affect the estimation of the other coefficients in a way that would change the interpretation of the results. Moreover, the interpretation of the coefficient for *Time-to-physician* was not considered as intuitive as the categorical explanatory variables. Thus, keeping Triage level in the model was considered to enhance the overall interpretability of the results. An overview of the parameter estimations resulting from the different models is presented below:

|  | **Mälarsjukhuset Eskilstuna - Regression coefficients (95 % Confidence intervals)** | | | | |
| --- | --- | --- | --- | --- | --- |
| Independent variable | **Full model** | **No collinear vars.** | **No imputation** | **2018 data only** | **2019 data only** |
| Age 0-17 | -8.3 (-10.1 – -6.6) * | -9.1 (-10.8 – -7.3) * | -15.1 (-17.0 – -13.1) * | -9.1 (-11.6 – -6.5) * | -7.4 (-9.8 – -5.1) * |
| 18-64 (reference) | 0 | 0 | 0 | 0 | 0 |
| 65-79 | 10.3 (8.6 – 12.1) * | 10.5 (8.8 – 12.2) * | 9.6 (7.7 – 11.4) * | 8.2 (5.7 – 10.7) * | 12.5 (10.1 – 14.9) * |
| 80+ | 11.3 (9.2 – 13.5) * | 11.8 (9.6 – 13.9) * | 12.4 (10.1 – 14.7) * | 8.5 (5.4 – 11.7) * | 14.0 (11.0 – 17.0) * |
| Sex, female | 2.7 (1.4 – 3.9) * | 3.0 (1.7 – 4.2) * | 5.2 (3.9 – 6.6) * | 3.6 (1.8 – 5.4) * | 1.8 (0.1 – 3.5) |
| Arrival day |  |  |  |  |  |
| Monday | 0.5 (-1.3 – 2.2) | 0.5 (-1.3 – 2.3) | 0.6 (-1.4 – 2.5) | 2.2 (-0.4 – 4.8) | -1.3 (-3.7 – 1.2) |
| Tue-Fri (ref.) | 0 | 0 | 0 | 0 | 0 |
| Weekend | 0.8 (-0.6 – 2.3) | 0.8 (-0.6 – 2.3) | 1.9 (0.3 – 3.5) | 1.5 (-0.6 – 3.6) | 0.1 (-1.9 – 2.2) |
| Time of day |  |  |  |  |  |
| 8 AM-4:59 PM (ref.) | 0 | 0 | 0 | 0 | 0 |
| 5 PM-9:59 PM | -6.8 (-8.3 – -5.3) * | -6.6 (-8.1 – -5.1) * | -5.5 (-7.2 – -3.9) * | -5.7 (-7.9 – -3.6) * | -7.8 (-9.9 – -5.6) * |
| 10 PM-7:59 AM | -10.2 (-11.9 – -8.5) * | -9.6 (-11.4 – -7.9) * | -9.0 (-11.0 – -7.1) * | -7.9 (-10.5 – -5.4) * | -12.2 (-14.6 – -9.8) * |
| Arrival by ambulance | 19.8 (18.0 – 21.6) * | 17.2 (15.4 – 19.0) * | 17.6 (15.7 – 19.5) * | 20.1 (17.5 – 22.7) * | 19.6 (17.1 – 22.2) * |
| Referral | 5.7 (1.3 – 10.1) | 6.7 (2.2 – 11.1) * | 3.4 (-1.3 – 8.0) | -14.5 (-24.5 – -4.5) * | 9.5 (4.5 – 14.4) * |
| Crowding |  |  |  |  |  |
| Low 0-75% (ref.) | 0 | 0 | 0 | 0 | 0 |
| Moderate 76-94% | 9.6 (8.0 – 11.1) * | 9.2 (7.7 – 10.8) * | 10.4 (8.7 – 12.1) * | 7.8 (5.5 – 10.1) * | 11.2 (9.0 – 13.3) * |
| High 95-100% | 11.5 (8.8 – 14.3) * | 11.3 (8.5 – 14.1) * | 12.8 (9.8 – 15.8) * | 9.0 (4.8 – 13.2) * | 13.4 (9.7 – 17.0) * |
| Triage level |  |  |  |  |  |
| Red (ref.) | 0 | -^c^ | 0 | 0 | 0 |
| Orange | 64.2 (60.0 – 68.4) * | -^c^ | 59.0 (54.3 – 63.6) * | 60.2 (54.1 – 66.4) * | 68.2 (62.3 – 74.1) * |
| Yellow | 65.8 (61.5 – 70.0) * | -^c^ | 63.0 (58.4 – 67.7) * | 60.5 (54.4 – 66.6) * | 70.9 (65.0 – 76.7) * |
| Green | 57.7 (53.3 – 62.2) * | -^c^ | 51.2 (46.4 – 56.1) * | 51.9 (45.4 – 58.4) * | 62.8 (56.7 – 68.9) * |
| Blue | 52.6 (47.8 – 57.3) * | -^c^ | 36.8 (31.6 – 42.0) * | 49.9 (43.1 – 56.7) * | 55.9 (49.2 – 62.6) * |
| Time-to-Physician ^a^ | 0.9 (0.9, 0.9) * | 0.9 (0.9 – 0.9) * | 0.9 (0.9 – 0.9) * | 0.9 (0.9 – 0.9) * | 0.9 (0.9 – 0.9) * |
| Laboratory analysis |  |  |  |  |  |
| No labs done (ref.) | 0 | 0 | 0 | 0 | 0 |
| Labs, excl. TnI&D-Di | 59.6 (58.0 – 61.3) * | 62.7 (61.1 – 64.2) * | 44.5 (42.6 – 46.3) * | 60.8 (58.4 – 63.2) * | 58.8 (56.6 – 61.1) * |
| Labs, incl. TnI | 61.8 (59.2 – 64.5) * | 64.9 (62.3 – 67.4) * | 43.8 (41.0 – 46.6) * | 62.2 (58.3 – 66.0) * | 61.6 (57.9 – 65.2) * |
| Labs, incl. D-Di | 68.3 (62.7 – 74.0) * | 71.3 (65.6 – 76.9) * | 53.4 (47.6 – 59.3) * | 65.7 (57.9 – 73.4) * | 72.9 (64.6 – 81.2) * |
| Labs incl. TnI&D-Di | 98.7 (92.8 – 104.6) * | 102.5 (96.5 – 108.4) * | 81.6 (75.4 – 87.7) * | 93.9 (85.8 – 101.9) * | 105.7 (96.9 – 114.5) * |
| POC ^b^ analysis only | 26.0 (23.3 – 28.8) * | 27.7 (25.0 – 30.4) * | 19.4 (16.3 – 22.5) * | 24.4 (20.1 – 28.7) * | 26.9 (23.4 – 30.5) * |
| Diagnostic imaging |  |  |  |  |  |
| No radiology (ref.) | 0 | 0 | 0 | 0 | 0 |
| X-ray | 63.8 (62.1 – 65.5) * | 61.1 (59.4 – 62.7)* | 59.6 (57.7 – 61.4) * | 62.1 (59.6 – 64.6) * | 65.0 (62.7 – 67.3) * |
| Ultrasound | 121.2 (117.4 – 125.1)* | 122.8 (118.9 – 126.7)* | 119.1 (115.2 – 123.1) * | 119.2 (113.6 – 124.9)* | 122.9 (117.7 – 128.2)* |
| CT | 125.3 (123.5 – 127.1)* | 124.7 (122.8 – 126.5)* | 121.5 (119.6 – 123.5) * | 126.1 (123.5 – 128.8)* | 124.4 (121.9 – 127.0)* |
| Other examination^c^ | -^c^ | -^c^ | -^c^ | -^c^ | -^c^ |
| Intra-ED zone transfer | 46.4 (43.0 – 49.8) * | 46.4 (43.0 – 49.8) * | 44.1 (40.5 – 47.6) * | 47.6 (42.8 – 52.5) * | 45.1 (40.4 – 49.8) * |
| Disposition |  |  |  |  |  |
| Discharged (ref.) | 0 | 0 | 0 | 0 | 0 |
| Admitted, <85 % | 0.7 (-3.4 – 4.7) | -0.8 (-4.8 – 3.3) | 10.8 (6.4 – 15.3) * | -4.7 (-10.3 – 1.0) | 7.3 (1.4 – 13.1) |
| Admitted, 85-95 % | 4.5 (2.4 – 6.7) * | 2.6 (0.5 – 4.7) | 14.5 (12.1 – 16.8) * | 4.2 (1.2 – 7.2) * | 5.1 (2.1 – 8.1) * |
| Admitted, >95 % | 16.5 (14.3 – 18.8) * | 14.4 (12.2 – 16.6) * | 28.8 (26.3 – 31.2) * | 19.6 (16.3 – 22.9) * | 13.9 (10.9 – 16.9) * |
| Intercept | 15.4 (10.9 – 20.0) * | 75.1 (73.3 – 76.9) * | 38.0 (32.9 – 43.0) * | 18.3 (11.7 – 24.9) * | 12.2 (5.8 – 18.5) * |

* = Statistically significant association with the dependent variable, p-value < 0.01
^a^ = Continuous variable, unit = 1 minute increments
^b^= Point-of-care
^c^= Omitted because of collinearity. No “Other examinations” (i.e. MRI, fluoroscopy angiography etc.) were carried out during ED stay at MSE

|  | **Örebro university hospital – Regression coefficients (95 % Confidence intervals)** | | | | |
| --- | --- | --- | --- | --- | --- |
| Independent variable | **Full model** | **No collinear vars.** | **No imputation** | **2018 data only** | **2019 data only** |
| Age 0-17 | 11.8 (10.6 – 13.0) * | 14.2 (13.0 – 15.4) * | 9.7 (8.4 – 11.1) * | 9.5 (7.8 – 11.2) * | 13.9 (12.2 – 15.6) * |
| 18-64 (reference) | 0 | 0 | 0 | 0 | 0 |
| 65-79 | 7.6 (6.3 – 9.0) * | 8.3 (7.0 – 9.7) * | 8.7 (7.3 – 10.1) * | 7.6 (5.7 – 9.5) * | 7.6 (5.7 – 9.5) * |
| 80+ | 14.7 (13.1 – 16.4) * | 15.2 (13.5 – 16.9) * | 15.5 (13.8 – 17.3) * | 14.9 (12.5 – 17.2) * | 14.5 (12.2 – 16.9) * |
| Sex, female | 3.0 (2.1 – 3.9) * | 3.0 (2.0 – 3.9) * | 3.5 (2.5 – 4.5) * | 2.6 (1.3 – 3.9) * | 3.3 (2.0 – 4.6) * |
| Arrival day |  |  |  |  |  |
| Monday | 2.0 (0.7 – 3.3) * | 2.1 (0.7 – 3.4) * | 2.4 (1.0 – 3.9) * | 1.5 (-0.4 – 3.3) | 2.5 (0.6 – 4.4) |
| Tue-Fri (ref.) | 0 | 0 | 0 | 0 | 0 |
| Weekend | -5.2 (-6.3 – -4.2) * | -5.4 (-6.5 – -4.3) * | -6.3 (-7.4 – -5.1) * | -4.3 (-5.8 – -2.8) * | -6.2 (-7.7 – -4.6) * |
| Time of day |  |  |  |  |  |
| 8 AM-4:59 PM (ref.) | 0 | 0 | 0 | 0 | 0 |
| 5 PM-9:59 PM | -12.6 (-13.7 – -11.5) * | -11.9 (-13.0 – -10.8) * | -13.9 (-15.1 – -12.7) * | -13.3 (-14.9 – -11.8) * | -11.9 (-13.5 – -10.3) * |
| 10 PM-7:59 AM | -13.8 (-15.1 – -12.6) * | -12.4 (-13.7 – -11.1) * | -14.3 (-15.7 – -12.9) * | -14.7 (-16.4 – -12.9) * | -13.1 (-14.9 – -11.3) * |
| Arrival by ambulance | 19.3 (17.9 – 20.7) * | 19.6 (18.3 – 20.9) * | 18.0 (16.5 – 19.4) * | 18.0 (16.0 – 19.9) * | 20.4 (18.5 – 22.3) * |
| Referral | 2.9 (1.1 – 4.6) * | 2.0 (0.3 – 3.8) | 1.8 (0.0 – 3.7) | 1.7 (-0.7 – 4.0) | 4.2 (1.6 – 6.7) * |
| Crowding |  |  |  |  |  |
| Low 0-75% (ref.) | 0 | 0 | 0 | 0 | 0 |
| Moderate 76-94% | 5.4 (4.2 – 6.6) * | 5.6 (4.4 – 6.8) * | 5.5 (4.2 – 6.7) * | 5.0 (3.3 – 6.6) * | 6.0 (4.3 – 7.7) * |
| High 95-100% | 9.8 (7.6 – 11.9) * | 9.7 (7.4 – 11.9) * | 10.1 (7.7 – 12.4) * | 9.6 (6.7 – 12.4) * | 10.7 (7.3 – 14.0) * |
| Triage level |  |  |  |  |  |
| Red (ref.) | 0 | -^c^ | 0 | 0 | 0 |
| Orange | 52.1 (49.6 – 54.7) * | -^c^ | 53.5 (50.9 – 56.2) * | 53.5 (49.9 – 57.1) * | 51.0 (47.4 – 54.6) * |
| Yellow | 38.6 (36.1 – 41.2) * | -^c^ | 43.4 (40.8 – 46.1) * | 40.5 (36.9 – 44.1) * | 36.8 (33.2 – 40.4) * |
| Green | 30.0 (27.2 – 32.7) * | -^c^ | 32.3 (29.4 – 35.2) * | 30.8 (26.9 – 34.7) * | 29.3 (25.4 – 33.2) * |
| Blue | 19.0 (16.0 – 22.1) * | -^c^ | 15.3 (12.1 – 18.6) * | 22.9 (18.7 – 27.1) * | 14.4 (9.9 – 18.9) * |
| Time-to-Physician ^a^ | 0.9 (0.9, 0.9) * | 0.9 (0.9 – 0.9) * | 0.9 (0.9 – 0.9) * | 0.9 (0.9 – 0.9) * | 0.9 (0.9 – 0.9) * |
| Laboratory analysis |  |  |  |  |  |
| No labs done (ref.) | 0 | 0 | 0 | 0 | 0 |
| Labs, excl. TnI&D-Di | 61.1 (59.9 – 62.4) * | 66.3 (65.1 – 67.5) * | 55.0 (53.7 – 56.4) * | 61.0 (59.2 – 62.8) * | 61.2 (59.5 – 63.0) * |
| Labs, incl. TnI | 52.8 (51.3 – 54.4) * | 58.5 (57.0 – 60.1) * | 44.8 (43.1 – 46.5) * | 51.3 (49.1 – 53.5) * | 54.4 (52.2 – 56.6) * |
| Labs, incl. D-Di | 83.3 (76.9 – 89.7) * | 85.7 (79.3 – 92.2) * | 76.6 (70.0 – 83.2) * | 82.3 (73.2 – 91.4) * | 84.2 (75.1 – 93.2) * |
| Labs incl. TnI&D-Di | 98.3 (93.5 – 103.1) * | 104.8 (99.9 – 109.6) * | 90.2 (85.3 – 95.1) * | 100.4 (93.5 – 107.2) * | 96.3 (89.5 – 103.2) * |
| POC ^b^ analysis only | 38.8 (36.5 – 41.1) * | 43.5 (41.2 – 45.8) * | 34.0 (31.5 – 36.4) * | 40.7 (37.7 – 43.7) * | 37.5 (33.8 – 41.1) * |
| Diagnostic imaging |  |  |  |  |  |
| No radiology (ref.) | 0 | 0 | 0 | 0 | 0 |
| X-ray | 67.7 (66.0 – 69.4) * | 63.5 (61.8 – 65.1) * | 70.0 (68.2 – 71.8) * | 72.7 (70.3 – 75.1) * | 62.9 (60.5 – 65.3) * |
| Ultrasound | 148.5 (145.0 – 152.0) * | 150.3 (146.7 – 153.8) * | 148.5 (144.8 – 152.1) * | 148.9 (143.9 – 153.8) * | 148.3 (143.3 – 153.3) * |
| CT | 131.7 (130.3 – 133.1) * | 133.3 (131.9 – 134.7) * | 133.9 (132.4 – 135.3) * | 130.3 (128.3 – 132.3) * | 133.0 (131.1 – 135.0) * |
| Other examination^c^ | 143.1 (132.8 – 153.4) * | 143.0 (132.6 – 153.4) * | 148.1 (136.6 – 159.6) * | 143.4 (128.9 – 157.8) * | 142.9 (128.1 – 157.6) * |
| Intra-ED zone transfer | 93.5 (90.9 – 96.1) * | 95.1 (92.5 – 97.7) * | 95.4 (92.2 – 98.6) * | 95.3 (91.6 – 98.9) * | 91.9 (88.2 – 95.6) * |
| Disposition |  |  |  |  |  |
| Discharged (ref.) | 0 | 0 | 0 | 0 | 0 |
| Admitted, <85 % | 3.5 (1.5 – 5.6) * | 3.5 (1.4 – 5.6) * | 5.2 (3.0 – 7.4) * | 6.5 (3.6 – 9.3) * | 0.6 (-2.5 – 3.6) |
| Admitted, 85-95 % | 8.0 (6.5 – 9.4) * | 7.9 (6.4 – 9.3) * | 10.0 (8.5 – 11.5) * | 10.2 (8.2 – 12.3) * | 5.8 (3.7 – 7.9) * |
| Admitted, >95 % | 7.4 (5.2 – 9.7) * | 7.0 (4.7 – 9.3) * | 10.6 (8.2 – 13.0) * | 9.4 (6.2 – 12.6) * | 5.5 (2.3 – 8.7) * |
| Intercept | 28.2 (25.3 – 31.0) * | 59.7 (58.4 – 61.1) * | 35.6 (32.6 – 38.6) * | 25.7 (21.7 – 29.8) * | 30.4 (26.4 – 34.5) * |

* = Statistically significant association with the dependent variable, p-value < 0.01
^a^ = Continuous variable, unit = 1-minute increments
^b^= Point-of-care
^c^= Advanced diagnostic imaging excl. CT – e.g. Magnetic resonance imaging (MRI) or fluoroscopy

1. Carpenter J, Kenward M. Multiple Imputation and its Application. 1. Aufl. ed. Newark: Wiley; 2012.

2. Canette I, Marchenko Y. Combining results other than coefficients in e(b) with multiply imputed data. StataCorp.

3. Bowerman BL, O'Connell RT, Murphree ES. Regression analysis : unified concepts, practical applications, and computer implementation. First edition. ed. New York, New York (222 East 46th Street, New York, NY 10017): Business Expert Press; 2015.
